# Association mapping of drought tolerance-related traits in barley to complement a traditional biparental QTL mapping study

**DOI:** 10.1007/s00122-017-2994-1

**Published:** 2017-10-25

**Authors:** Magdalena Wójcik-Jagła, Anna Fiust, Janusz Kościelniak, Marcin Rapacz

**Affiliations:** Department of Plant Physiology, University of Agriculture in Krakow, Podłużna 3, 30-239 Kraków, Poland

## Abstract

**Key message:**

**Association mapping of drought-related traits in barley was used to increase the density of existing QTL maps without recreating mapping populations.**

**Abstract:**

We used 109 spring barley genotypes exhibiting high or low drought tolerance to elucidate the associations between diversity array technology sequencing (DArTseq) and single nucleotide polymorphism (SNP) markers and various physiological parameters related to plant responses to drought conditions. The study was performed in controlled conditions (growth chambers), drought tolerance was phenotyped in the four-leaf seedlings. We identified 58 associations including 34 new markers (i.e., 16 DArTseq and 18 SNP markers). The results for three markers were consistent with the data obtained in an earlier traditional biparental QTL mapping study. The regions neighboring markers on linkage group 2H contained the highest number of significant marker–trait associations. Five markers related to the photosynthetic activity of photosystem II were detected on chromosome 4H. The lowest number of associations were observed for the sequences neighboring DArT markers on linkage group 6H. A chromosome 3H region related to water use efficiency and net photosynthesis rate in both biparental QTL, and association study, may be particularly valuable, as these parameters correspond to the ability of plants to remain highly productive under water deficit stress. Our findings confirm that association mapping can increase the density of existing QTL maps without recreating mapping populations.

**Electronic supplementary material:**

The online version of this article (doi:10.1007/s00122-017-2994-1) contains supplementary material, which is available to authorized users.

## Introduction

Analyzing quantitative traits is challenging, which makes these traits difficult to improve via plant breeding. Therefore, for many years, research tended to focus on the identification of single genes with the biggest effects on the overall phenotype until two methods enabling investigations of quantitative traits have been developed—genetic linkage-based biparental QTL mapping and association mapping. Both of these approaches rely on the analysis of the strength of the relationships between genetic markers and phenotypic traits (Korte and Farlow 2013). However, while QTL mapping uses correlations between the sharing of chromosomal regions among relatives and their trait similarities (as it is a highly controlled experiment, in which the level of kinship is known), association mapping directly combines the genotype and phenotype in a “natural” experiment, where the relationships between the objects are not controlled (Myles et al. 2009; Purcell et al. 2003).

Association mapping, which is also called linkage disequilibrium (LD) mapping or population mapping, is based on the non-accidental associations of alleles at different loci on the same chromosome (Mackay and Powell 2007; Flint-Garcia et al. 2003). In contrast to QTL analyses, association mapping uses plant materials that are not closely related (i.e., natural populations) (Korte and Farlow 2013). Using LD to map quantitative traits is more challenging than the standard QTL analysis protocol. However, there are potential advantages, including that it enables a more precise determination of the location of a QTL associated with a specific trait. Furthermore, association mapping is very effective for studying species that are difficult to cross or clone, or those with a long reproduction time (Nordborg and Weigel 2008). Investigations based on LD analyses and association mapping have been conducted for many species, including monocots such as corn, barley, rice and sorghum (Zhu et al. 2008; Korte and Farlow 2013). In barley plants, associations have been detected between genetic markers and the following phenotypic traits: flowering time (Stracke et al. 2009), yield (Kraakman et al. 2004; Gawenda et al. 2015), disease resistance (Roy et al. 2010; Massman et al. 2011), drought tolerance (Varshney et al. 2012), salinity tolerance (Fan et al. 2016) and freezing tolerance (Rapacz et al. 2010a; Visioni et al. 2013). An essential flaw of association mapping is that there is a risk of obtaining false positive results (Pritchard et al. 2000) where LD is a result of existence of subpopulations, not true associations (Cardon and Bell 2001). This is a result of using an open, natural experimental system, in which it is hard to find out about the frequency of recombination events (Myles et al. 2009). However, there are several methods involving analyses of the population structure that can be used to ensure the obtained results correspond to real effects (Porras-Hurtado et al. 2013).

Nowadays the best results are obtained by combining association mapping and biparental QTL mapping. Association mapping in some particular cases may be insufficient to detect an association (low frequency alleles, QTL with small effects), and then biparental QTL mapping can be used as a way to control the population structure and recombination frequency (Myles et al. 2009). There have been statistical methods developed to connect those two approaches, namely joint linkage-association mapping as first (Wu and Zeng 2001; Meuwissen et al. 2002), and, more recent and complex, nested association mapping (NAM) (Yu et al. 2008; McMullen et al. 2009).

**Fig. 1:**
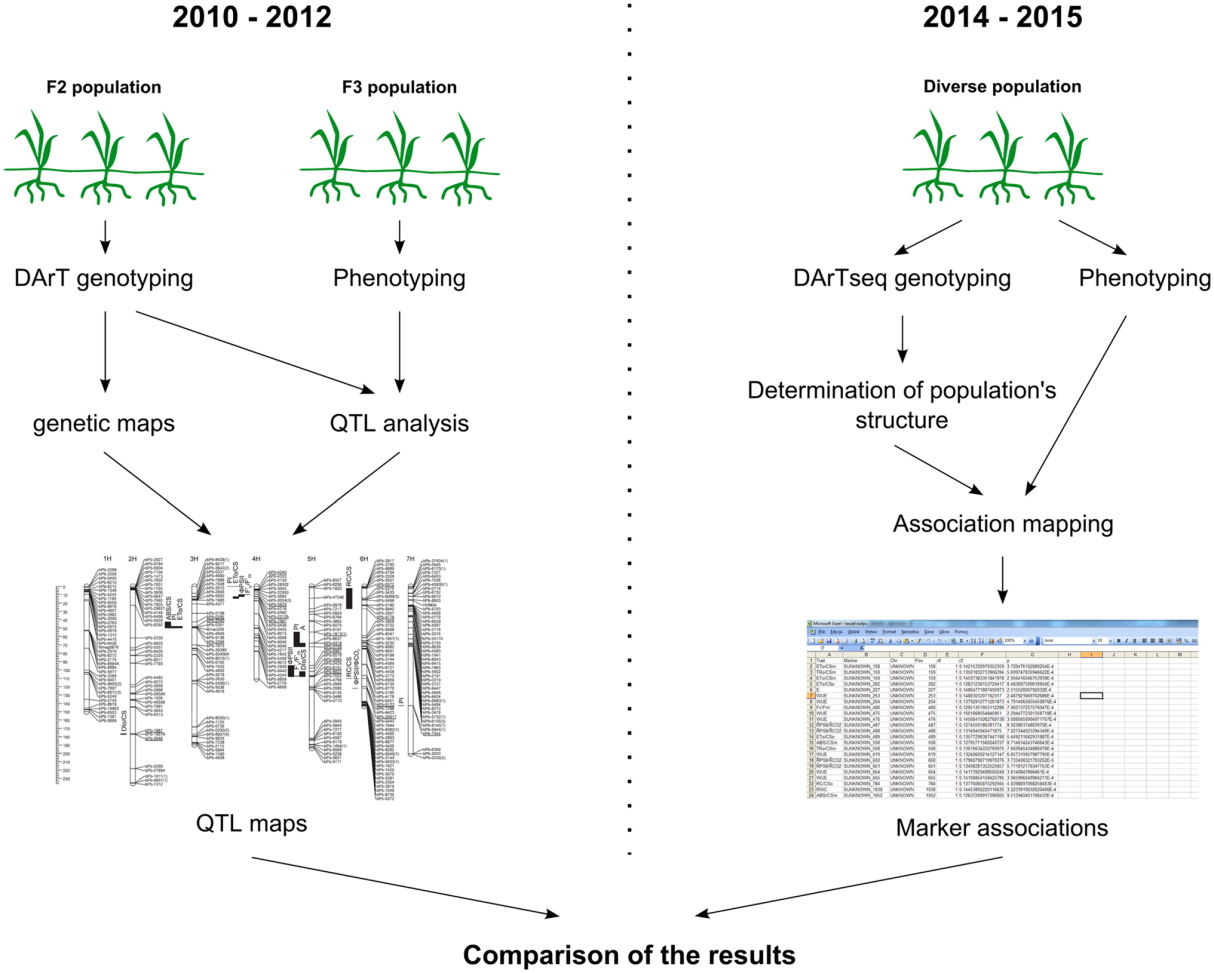
The experiments’ outline. Results from the 2010–2012 experiments were published in Wójcik-Jagła et al. (2013). Experiments from the years 2014–2015 and their results are described in the present study

The aim of this study was to thoroughly investigate the genetic determinants of drought tolerance in Polish barleys by combining two approaches to studying quantitative traits. In a previous study, we conducted a QTL analysis on two barley F_2_ populations (Wójcik-Jagła et al. 2013). To complement this approach and confirm previously obtained results in the present study, we performed an association mapping experiment using a population of barley cultivars and advanced breeding materials (Fig. 1). The second and more practical purpose of this study was to saturate the QTL regions revealed in our previous study (Wójcik-Jagła et al. 2013) with new DArTseq and SNP markers.

## Materials and methods

### Plant materials

Barley materials consisted of advanced breeding lines and cultivars grown by the following two breeding companies: HR Strzelce Ltd. group IHAR (located in Strzelce, Poland—527 genotypes) and HR Danko sp. z o.o. (located in Choryń, Poland—180 genotypes). We also used doubled-haploid lines (506 genotypes) developed by HR Strzelce from ten crossings between “Suweren” (drought-tolerant cultivar) and other cultivars with different origins.

### Selection of genotypes for association studies

The selection of barley genotypes of potential high and low drought tolerance was performed basing on specific sequence-tagged site markers and simple sequence repeat markers derived from diversity array technology (DArT) markers (Fiust et al. 2015). The markers were associated with two QTL regions reported in Wójcik-Jagła et al. (2013), related to chlorophyll *a* fluorescence parameters, as well as with additional seven regions significant in the preliminary simple marker regression analysis, related to electrolyte leakage (EL), water content (WC), net photosynthesis rate (*P*_n_), and rate of PSII quantum efficiency related to the quantum efficiency of CO_2_ assimilation (ΦPSII/ΦCO_2_).

The youngest leaves on field-grown plants were collected just before heading, after which they were frozen in liquid nitrogen and lyophilized (Freezone 4.5, Labconco, Kansas City, MO, USA). The lyophilized leaves were ground to powder, from which DNA was extracted using the GeneJET Plant Genomic DNA Purification Mini Kit (Thermo Scientific, Waltham, USA). The quantity and quality of purified DNA were assessed using the Q500 spectrophotometer (Quawell, San Jose, USA). The DNA from the 1213 tested genotypes was then used for polymerase chain reaction (PCR) amplifications. The PCRs were conducted with 19 primer combinations (Table 1). Each PCR consisted of the following components: 1 × PCR buffer with (NH_4_)_2_SO_4_ (Thermo Scientific), 250 nM each primer, 3.5 mM MgCl_2_, 200 mM dNTP, 0.4 U Taq polymerase (Thermo Scientific), 20 ng DNA, and 400 μM spermidine (Sigma-Aldrich, Darmstadt, Germany). The marker sequences were amplified in a Labcycler (SensoQuest, Göttingen, Germany) using one of three temperature profiles depending on primer melting temperatures (Table 2).

**Table 1:** DArT-derived markers used for the genomic selection for drought tolerance, type of marker, amplification profile name (see Table 2) and primer sequences

Marker	Marker type (amplification profile)	Forward (5′–3′)	Reverse (5′–3′)
bPb-1312	STS (52)	TGAAACATCGAAACCCACAA	CTCCATTCCTCTGGCTATGC
bPb-8884	STS (55)	CATGTGCAAACTGTCCCAAC	CTAGCAGCAGCAAGTGCATC
bPb-1967	STS (55)	AGGTTTTCAAGCAGCTACGC	CAAGAAAGCAGATGGCACAA
bPb-1967b	STS (55)	TTGCAGAAGGCGGATAATTC	TTTCGGGCACTGATTTCAAC
bPb-0858	STS (58)	GGCAGGTACACCGCCACT	TCAGAGCACACGTATGCAGAT
bPb-2040b	STS (55)	CCATAAAGTTAAGAATTTGCCTCA	GCAACTCACACCCCTTCTGT
bPb-6399	STS (55)	TGCACAGCCTAAAAGAATCG	TGTTGGCACAGCATGTTAGC
bPb-6450	STS (52)	ACGCCCAAGTCACAAATCTT	GGTCCAGTTCCTGTTCTTGG
bPb-0994	STS (52)	CCACCCCAATGTGTTCTCTC	TGCAGGCGAAAATTGTTGTA
bPb-1051	STS (55)	CGTCCCCATGATCCTTTTT	GCAGGCTATTTTGTGGCTTT
bPb-1051b	STS (55)	CGGGAAGCTCTATCACTCGT	TGATATGTGCAGCGTCCATT
bPb-8589	STS (55)	AGCTCTCTGTAGATCAGGTTGC	CGACAACGGGAATGGAAC
bPb-9645	STS (55)	CATGTCAAAAGCTATGGATGC	CTTGCCCTCTCTCGTCAAAC
bPb-6735	STS (52)	TCAGGCATCTGCAATTTTTG	TTCGGTCCTTCTTGCATACC
bPb-6721	STS (55)	GGAAAAACAAAACTGAGGCAAA	GTGGATTGTGAGGCCGATT
bPb-3908	STS (52)	TCGAGATGCATCAGACTTTCA	TTCGGTCCTTCTTGCATACC
bPb-7786	STS (52)	GCTGGAGACTTGGAGGACAG	TGGTTATTACCACAACCAGA
Bmag0876	SSR (55)	AATTAAAAGCTGAAGGTCTACA	CTGCTCCTTCAACGACTAC
Bmac209	SSR (55)	ATGCCTGTGTGTGGACCAT	CTAGCAACTTCCCAACCGAC
scssr02503	SSR (55)	AACAACTTTTGATGGACAAACC	TGTCTTTTCTTTTTGCTCTGC

**Table 2:** Amplification profiles

Cycle signature	52	55	58	Time (s)	Number of cycles
Parameters	temp. (°C)	temp. (°C)	temp. (°C)		
Pre-denaturation	95	95	95	120	1
Denaturation	94	94	94	45	7
Primer linkage	59	62	65	45	
Polymerase linkage	72	72	72	45	
Denaturation	94	94	94	45	41
Primer linkage	52	55	58	45	
Polymerase linkage	72	72	72	45	
Annealing	72	72	72	600	1

### Plant growth and drought treatment

Based on the molecular selection results, 109 lines exhibiting low or high drought tolerance were chosen (Online Resource 1). Seeds of these genotypes were sown in pots (5 dm^3^, one genotype per pot and one pot per genotype, 12 seeds per genotype) filled with a mixture of universal garden soil substrate (Ekoziem, Jurkow, Poland) and sand (1:1, v:v) and put in growth chambers in a fully randomized order. Genotypes were sown sequentially (10 genotypes per day). The plants were grown incubated in 25 °C/17 °C (day/night), with a 14-light/10-h dark photoperiod (irradiance: 400 μmol m^−2^ s^−1^; HPS lamps, SON-T+ AGRO, Philips, Brussels, Belgium). The plants were watered as required and treated once a week with Florovit multipurpose fertilizer (Inco, Góra Kalwaria, Poland) according to the manufacturer’s instructions. The soil water content (WC) was maintained at 70% maximum water capacity (MWC) until plants reached the four-leaf stage by adding the required amount of water based on daily weight measurements. Water was subsequently withheld from drought-treated plants for 10 days, during which the MWC gradually decreased to 21% according to the HydroSense Soil Water Content Measurement System (Campbell Scientific, Thuringowa Central, Australia). Under these conditions, leaves of all genotypes exhibited symptoms of turgor loss. Soil WC was kept at 70% MWC in the not treated (control) plants until the measurements of the physiological parameters.

### Genotyping

We extracted DNA from the fourth leaf (i.e., youngest) of the selected 109 genotypes, and assessed the DNA quality using the same kit and quality assessment procedure described above. The DNA samples were then screened using DArT sequencing (DArTseq) technology (http://www.diversityarrays.com/dart-application-dartseq), which combines DArT technology with next-generation sequencing. The polymorphism was identified in a 0/1 system, which indicates either the presence or absence of a marker.

### Phenotyping

Due to diurnal changes observed usually for many of measured parameters all the measurements were made at midday (between 11 a.m. and 2 p.m.). Photosystem II (PSII) activity, gas exchange, relative WC (RWC), and electrolyte leakage were measured in plants with 21% MWC. The PSII photochemical activity was measured in the middle part of the second leaf using eight replicates (leaves from 8 different plants randomly chosen out of 12 growing in the same pot) per genotype. We used two measurement systems as previously described, namely the modulated fluorescence system FMS2 (Hansatech, Kings Lynn, UK) and the fast chlorophyll fluorescence induction kinetics fluorimeter Handy PEA (Hansatech, Kings Lynn, UK) (Rapacz et al. 2010a, b, Wójcik-Jagła et al. 2013). The FMS2 system was used to calculate the following parameters: PSII antenna trapping efficiency ($F_{v}^{\prime}/F_{m}^{\prime}$), photochemical light energy quenching coefficient (*q*_P_), and quantum yield of electron transport in PSII [i.e., ΦPSII = $(F_{m}^{\prime} - F_{s}^{})/F_{m}^{\prime}$] (Genty et al. 1989). The Handy PEA was used to analyze parameters based on the theory of energy flow in PSII, and involved the OJIP test (Strasser et al. 2000). The fluxes in the energy absorbed by PSII antennae (ABS), trapped in PSII reaction centers (TR), used for electron transport (ET), and dissipated from PSII, and dissipated from PSII (DI), as well as the maximum number of active reaction centers (RC) were calculated per excited leaf cross section (CS) at *t* = 0 (CS_0_) or at the *t* of *F*_m_ (CS_m_). The energy fluxes per active PSII RC were also considered. Additionally, the overall PSII photochemical performance index for equal absorption (PI_ABS_) was calculated together with the more traditional performance index of energy trapped in PSII (*F*_v_/*F*_m_). The middle part of the second leaf was used to measure gas exchange parameters with a Ciras-3 infrared gas analyzer (PP Systems, Hitchin, UK) and a Parkinson leaf chamber (PLC6) as described elsewhere (Rapacz et al. 2010a). The controlled environmental conditions were as follows: 400 μmol mol^−1^ CO_2_, 30% relative humidity, 500 µmol m^−2^ s^−1^ irradiance, and 25 °C leaf temperature. The following parameters were measured based on gas exchange and chlorophyll fluorescence data for 10 replicates (leaves from 10 different plants randomly chosen out of 12 growing in the same pot): transpiration rate (*E*), *P*_n_, intercellular CO_2_ concentration (*C*_i_), stomatal conductivity (*g*_s_), quantum yield of CO_2_ assimilation (ΦCO_2_), water use efficiency (WUE), and ΦPSII/ΦCO_2_. The RWC was measured for the first (i.e., the oldest) leaf for eight replicates (leaves from 8 different plants randomly chosen out of 12 growing in the same pot) per genotype. The samples were weighed (fresh weight; FW), placed in 25-ml closed tubes filled with water, and shaken in darkness for 24 h. Samples were weighed again to determine the turgor weight (TW), after which they were transferred to paper envelopes and incubated at 70 °C in an air drying chamber (Lumel, Zielona Góra, Poland) for 48 h. The dry weight (DW) of the samples was then measured. The RWC was calculated using the following equation according to Barrs (1968):

$$ {\text{RWC }}(\% ) \, = \, ({\text{FW }} - {\text{ DW}}) \, \times \, ({\text{TW }} - {\text{ DW}})^{ - 1} \times { 1}00. $$

Membrane integrity was assessed according to electrolyte leakage for eight replicates (leaves from 8 different plants randomly chosen out of 12 growing in the same pot) per genotype. The measurements were conducted with 1-cm cuttings from the middle part of the first (i.e., oldest) leaf as described by Rapacz et al. (2010a).

### Determination of population structure

The physical positions of markers within the QTL regions for drought tolerance in spring barley (Wójcik-Jagła et al. 2013) were identified using the ViroBLAST program (Deng et al. 2007) and genome sequence databases for Morex, Barke, and Bowman cultivars as references. The marker sequences most similar to the contig sequence were considered. Based on the determined physical positions (Wójcik-Jagła et al. 2013), a set of DArTseq and single nucleotide polymorphism (SNP) markers within 5 cM were developed and subjected to association mapping. As the existence of substructures within a population can increase type I error in association analyses (Cardon and Bell 2001) we studied possible relationships between the genotypes using two methods. First, the similarities within the studied set of markers and genotypes were identified according to cluster analyses using the UPGMA method with Dice’s similarity coefficient. The structure of the tested datasets was determined, and repetitive results (i.e., identical polymorphism patterns) were eliminated. The analysis was performed using Statistica 10 software (Statsoft, Tulsa, USA). The detailed population structure analysis was completed using the STRUCTURE v. 2.3.4 program (Pritchard et al. 2000). Compared to other software dedicated for population analysis, STRUCTURE can easily manage different types of markers, including SNPs and dominant loci such as DArTseq markers (Porras-Hurtado et al. 2013). The admixture model was chosen with 10,000 cycles and 1000 repeats per cycle. The test was conducted ten times for several subpopulations (*K* = 1 − 6). The *K* parameter was determined as described by Evanno et al. (2005).

### Association analysis

The marker–trait associations were determined using the TASSEL program (Ithaca, New York; Bradbury et al. 2007). The following datasets were used for the analysis: genotyping results (0/1 matrix), phenotyping results for drought tolerance-related parameters (numerical), and population structure described above. The genotypic data required a minimum allele frequency of 0.05. As barley is a self-pollinating plant, the degree of kinship is on a highly controlled level in populations of known origin, such as the one used in this study. Therefore, no correction for kinship was included. In consequence, associations were calculated according to the general linear model, which finds the ordinary least squares solution for each marker-trait association (Bradbury et al. 2007). The threshold probability level was set at 0.001. An additive data analysis model was used. To retrieve all of the significant effects we decided not to add any additional factors to the linear model. Probability values from the GLM model were adjusted using false discovery rate (FDR) according to the procedure by Benjamini and Hochberg (1995).

## Results

### Genotyping

The DArTseq genotyping of 109 spring barley lines with diverse drought responses revealed 15,828 specific DArTseq markers, of which 10,652 sequences were localized in defined contigs of Morex, Barke, or Bosman barley varieties. A total of 5153 sequences generated from the DArTseq analysis were not unambiguously assigned to any defined barley genome region. The degree of polymorphism of identified sequences varied in the analyzed population. We determined that 1676 DArTseq sequences were polymorphic in all tested genotypes. The least effective polymorphism assessment based on the allelic readings and the ratio of the genotypes tested (i.e., 73–80%) was observed for 86 DArTseq sequences. The polymorphism information content (PIC) of 2063 marker sequences was no more than 18%, which indicated the markers were relatively unsuitable for distinguishing between genotypes. We observed that 1649 sequences had the highest PIC within the tested genotypes (i.e., 49–51%). Additionally, 1005 marker sequences that were absent in most genotypes were identified as the least polymorphic (PIC ≤ 10%). The effectiveness of the polymorphism identification within tested genotypes was over 99%.

The tested genotypes also contained 7829 sequences with a SNP. The SNPs were identified based on the following three allelic variants: 0 and 1 (i.e., homozygous allele pattern for the reference/target base variant) and 2 (i.e., heterozygotes presenting with a two-allele pattern). Accordingly, 4541 sequences were identified as homozygous (i.e., 58% of the total number of sequences).

### Phenotyping

**Fig. 2:**
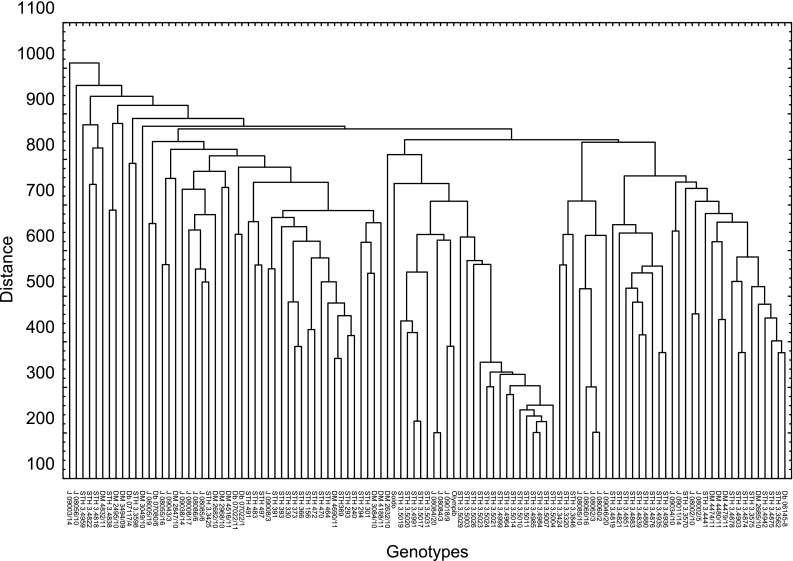
Results of the cluster analysis of the similarities within the studied set of genotypes using the UPGMA method with Dice’s similarity coefficient

The physiological parameters measured in this study were highly differentiated within the population (Online Resource 2). There were no significant differences between the plants in the control conditions (data not shown). The initial selection of drought tolerant and drought susceptible genotypes by means of molecular markers was quite successful and candidate tolerant genotypes performed better in drought. This difference was statistically significant in the case of key parameters of drought tolerance such as WC, RWC, *P*_n_ and *E*. In tolerant group, higher net assimilation rate in drought was thus accompanied by higher RWC which together with higher *E* suggested additional higher ability to water extraction from the soil. Among the studied parameters the highest differentiation between the genotypes was observed in the case of gas exchange related parameters (*P*_n_, *E*, *g*_s_, *C*_i_, WUE) as well as water relations and EL (Fig. 2). Out of all chlorophyll-fluorescence related parameters, PI(abs), which characterizes the overall PSII photochemical performance, varied most between genotypes.

### Association mapping

**Fig. 3:**
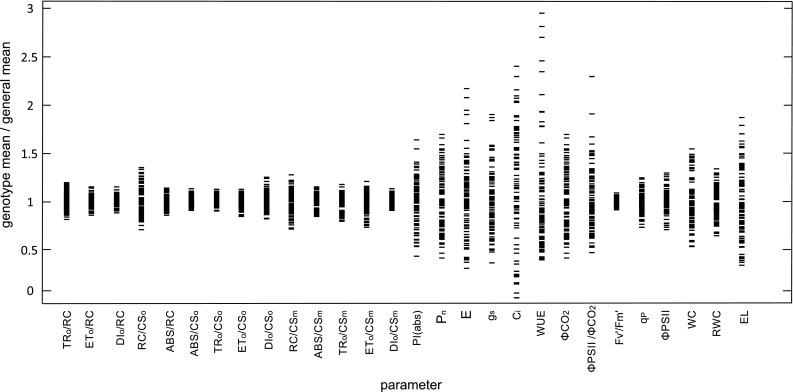
Variation in measured physiological characteristics of drought response among studied genotypes. Mean values for genotypes are normalized for general means

The genotyping data used for association mapping comprised 1001 DArTseq markers and 796 SNP markers located within 5 cM from DArT markers in regions with QTLs for drought tolerance-related parameters. The cluster analysis results did not reveal any subpopulations among the tested genotypes (Fig. 3). Three subpopulations were distinguished with the STRUCTURE program. The first included 26 genotypes, while the second and third subpopulations consisted of 32 and 51 breeding lines, respectively. Five genotypes (i.e., DB07022/1, DB07022/11, J08084/3, J08084/20, and STH34838) were highly likely to belong to more than one subpopulation (Table 3). The population’s structure was associated with drought tolerance to some extent. First subpopulation seemed to consist of mostly tolerant, while the third subpopulation—mostly susceptible to drought genotypes. But while the genotypes of the first subpopulation did have the highest level of water in their leaves, they also had the lowest WUE (Online Resource 2).

**Table 3:** Population’s structure obtained using STRUCTURE software

No.	Genotype	Probability that the genotype belongs to subpopulation			Subpopulation
		1S (p)	2S (p)	3S (p)	
1	DB06145-98	0	0	0.999	3
2	DM4832/11	0.105	0.227	0.668	3
3	DM4188/11	0.001	0.358	0.642	3
4	STH35004	1	0	0	1
5	STH35021	1	0	0	1
6	STH35026	1	0	0	1
7	STH240	0	1	0	2
8	DM3494/09	0.106	0.7	0.194	2
9	DM2847/10	0.536	0.372	0.092	1
10	DM4479/11	0	0.04	0.96	3
11	STH35007	1	0	0	1
12	STH35023	1	0	0	1
13	STH34942	0	0	1	2
14	STH393	0	1	0	2
15	DM3049/10	0.166	0.009	0.826	3
16	J08002/5	0.001	0.051	0.948	3
17	DM4480/11	0.001	0	0.999	3
18	STH35010	1	0	0	1
19	STH35024	1	0	0	1
20	STH470	0	1	0	2
21	STH391	0	0.909	0.091	2
22	J08002/10	0.072	0.05	0.878	3
23	DM4516/11	0.001	0.67	0.329	2
24	DM2682/10	0	0.703	0.297	2
25	STH35011	1	0	0	1
26	STH35029	0.924	0.067	0.009	1
27	STH366	0.001	0.999	0	2
28	Suweren	0	1	0	2
29	DM2495/10	0.25	0.239	0.51	3
30	OLYMPIC	0.417	0.398	0.185	1
31	DM3084/10	0.115	0.109	0.776	3
32	STH35014	1	0	0	1
33	STH35031	0.926	0.072	0.001	1
34	STH373	0.001	0.998	0.001	2
35	J09038/14	0,001	0.686	0.313	2
**36**	**DB07022/1**	**0.104**	**0.487**	**0.408**	**2/3**
37	DM2685/10	0.001	0	0.999	3
38	STH33562	0.001	0	0.999	3
39	STH35017	0.999	0	0	1
40	STH33570	0.404	0	0.595	3
41	STH497	0.086	0.619	0,295	2
42	J09046/20	0,02	0.253	0.727	3
43	DM4474/11	0,002	0.006	0.992	3
**44**	**DB07022/11**	**0.315**	**0.0.326**	**0.36**	**1/2/3**
45	STH34999	1	0	0	1
46	STH35019	0.911	0	0.089	1
47	STH33575	0	0	0.999	3
48	STH464	0.009	0.66	0.331	2
49	SOLDO	0.439	0.21	0.351	1
50	DB07080/6	0.296	0.055	0.649	3
51	STH35003	1	0	0	1
52	STH35020	1	0	0	1
53	STH33598	0.091	0	0.909	3
54	STH472	0.161	0.838	0	2
55	DM4690/11	0	1	0	2
56	J08060/2	0.004	0.219	0.777	3
57	J09003/14	0.185	0.332	0.482	3
58	STH33846	0.466	0.259	0.275	1
59	STH34936	0.001	0	0.999	3
60	STH34874	0.379	0	0.621	3
61	STH156	0.431	0.563	0.006	2
62	DB07117/4	0.058	0.411	0.531	3
63	J08060/16	0.01	0.21	0.78	3
64	J09008/3	0.005	0.323	0.672	3
65	STH33320	0.337	0,198	0.464	3
66	STH34984	1	0	0	1
67	STH34875	0	0	0.999	3
68	STH491	0.124	0.475	0.401	2
69	DM2632/10	0.562	0.186	0.252	1
70	J08062/3	0.006	0.216	0.778	3
71	J09011/14	0.258	0.092	0.651	3
72	STH33424	0.445	0.387	0.169	1
73	STH34985	1	0	0	1
74	STH34876	0.373	0	0.627	3
75	STH293	0.001	0.998	0.001	2
76	DM2968/10	0.012	0.762	0.226	2
**77**	**J08084/3**	**0.489**	**0.511**	**0**	**1/2**
78	J09018/9	0.385	0.445	0.169	2
79	STH33425	0.011	0.498	0.49	2
80	STH34818	0.045	0.194	0.761	3
81	STH34878	0.356	0	0.644	3
82	STH294	0.002	0.248	0.75	3
83	J08005/19	0.195	0.17	0.635	3
**84**	**J08084/20**	**0.489**	**0.511**	**0**	**1/2**
85	J09034/10	0.111	0.003	0.886	3
**86**	**STH34838**	**0.067**	**0.445**	**0.488**	**2/3**
87	STH34819	0.001	0.165	0.834	3
88	STH34880	0.398	0	0.601	3
89	STH301	0.005	0.239	0.756	3
90	J08008/17	0.001	0.585	0.415	2
91	J08085/6	0.001	0.571	0.429	2
92	J09043/3	0	0.001	0.999	3
93	STH34851	0.343	0.001	0.656	3
94	STH34821	0.293	0.061	0.645	3
95	STH34883	0.363	0	0.637	3
96	STH330	0	1	0	2
97	J08055/16	0	0.022	0.978	3
98	J08085/10	0.017	0.247	0.736	3
99	STH34991	0.999	0	0.001	1
100	STH34903	0.371	0	0.629	3
101	STH34822	0.439	0	0.561	3
102	STH34959	0.091	0.151	0.758	3
103	STH483	0.205	0.72	0.075	2
104	J08056/10	0.091	0.783	0.126	2
105	J08086/6	0.001	0.723	0.277	2
106	STH34441	0.001	0.538	0.461	2
107	STH34935	0.001	0	0.999	3
108	STH34839	0.385	0	0.615	3
109	STH34964	1	0	0	1

Genotypes probably belonging to more than one subpopulation are indicated in bold

The association mapping analysis uncovered 58 associations including 34 new markers (i.e., 16 DArTseq and 18 SNP markers). All of the associations were positively evaluated by FDR. The strength of the association between a marker and a phenotypic trait was assessed based on the probability value. The strongest relationship between a marker and a trait was observed for three DArTseq markers (i.e., 3986215, 3272729, and 4789498) as well as three SNP markers (i.e., 3255717, 5249538, and 100002239) (Table 4). Most of these sequences were significantly related to plant water relations parameters (e.g., RWC and WUE). Each of the revealed associations explained 6.97–14.52% of observed phenotypic variance in the studied traits.

**Table 4:**
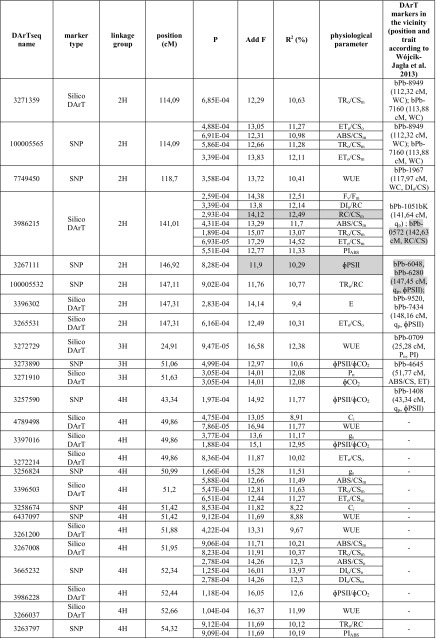

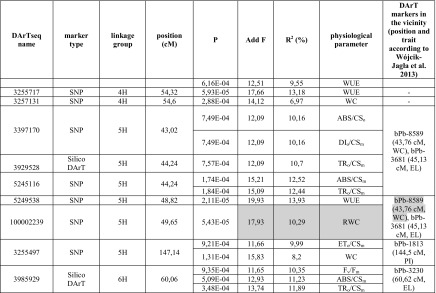
Markers associated with drought tolerance parameters in spring barley

*P* critical value of the probability of association of marker with the trait, *Add F F* value for the *F* test for additive effect of allele, *R* ^*2*^ percentages of phenotypic variance explained by individual marker, *F*_*v*_ */F*_*m*_ performance index of energy trapped in PSII, *q*_*P*_ photochemical light energy quenching coefficient, *ΦPSII* quantum yield of electron transport in PSII, *ABS* fluxes in the energy absorbed by PSII antennae, *CS*_*m*_ excited leaf cross section (CS) at the *t* of *F*_m_, *CS*_*o*_ excited leaf cross section (CS) at *t* = 0, *TR*_*o*_ fluxes in the energy trapped in PSII reaction centers, *ET*_*o*_ fluxes in the energy used for electron transport, *DI*_*o*_ fluxes in the energy dissipated from PSII, *RC* the maximum number of active reaction centers, *PI*_*ABS*_ the overall PSII photochemical performance index for equal absorption, *E* transpiration rate, *P*_*n*_ net photosynthesis rate, *C*_*i*_ intercellular CO_2_ concentration, *g*_*s*_ stomatal conductivity, *ΦCO*_*2*_ quantum yield of CO_2_ assimilation, *WUE* water use efficiency, *WC* water content, *RWC* relative water content, *EL* electrolyte leakage, consistent results of the both studies are shaded

The results for three markers were consistent with the observations of a previous study (Wójcik-Jagła et al. 2013; Table 4). Sequence 3986215 identified at position 141.01 cM on chromosome 2 was associated with many chlorophyll fluorescence-based characteristics of PSII performance in drought-stressed plants, including those related to photosynthetic energy conversion (i.e., ABS, TR, and ET) as well as the RC/CS. This sequence co-localized with the physical positions of markers bPb-0572 (142.63 cM) and bPb-1051bK flanking the QTL regions for *q*_P_ and RC/CS.

Similarly, marker 3267111 was associated with ΦPSII, and was located within 0.53 cM from markers bPb-6048 and bPb-6280 in *QPSII.sthf*-*2H* (Wójcik-Jagła et al. 2013). A comparable relationship was detected for markers 100002239 and bPb-8589 (*QWC.sthf*-*5H.2*; Wójcik-Jagła et al. 2013) from linkage group 5H (± 5.89 cM). These two markers were correlated with plant water relations parameters (e.g., RWC and WC).

The regions neighboring markers on linkage group 2H (Table 4) contained the highest number of significant marker–trait associations, particularly the 141.01–148.16 cM region, which included eight markers associated with 13 parameters related mostly to photosynthetic activity of drought-stressed plants. These results are similar to those observed in a previous QTL analysis (Wójcik-Jagła et al. 2013). We also detected a relatively high density of five markers related to PSII photosynthetic activity on chromosome 4H. Furthermore, the 43.34–53.6 cM region of this chromosome was rich in markers associated with parameters related to gas exchange in drought-stressed plants (Table 4). The lowest number of associations was observed for the sequences neighboring DArT markers on linkage group 6H (Table 4).

## Discussion

The idea of combining traditional biparental QTL mapping and association mapping in one analysis was first proposed almost 10 years ago in a review paper written by Myles et al. (2009). This new approach to QTL mapping was quickly implemented by Brachi et al. (2010) and Famoso et al. (2011) who aimed to uncover the genetic determinants of *Arabidopsis thaliana* flowering time in the field and characterize the genetic architecture of aluminum tolerance in rice, respectively. In these two studies, a traditional QTL analysis of mapping populations derived from biparental crosses was complemented with the association mapping of natural plant accessions. Unlike the study presented herein, these two approaches were designed as complementary from the beginning. The two earlier studies also used mapping populations that were created from selected cultivars of a set of natural accessions subjected to association mapping (Shindo et al. 2005; Zhao et al. 2011). In the present study, only one of the four parents included in the previous study (Wójcik-Jagła et al. 2013) was used for association mapping. Additionally, the barley materials used in this study and previously were substantially different (i.e., *F*_2_ mapping populations for the QTL study and a diverse collection of advanced breeding lines mixed with some cultivars grown in Poland). However, they were also similar because their gene pools were optimized for cultivation under the humid continental climate of Poland.

Combining the results from previous traditional QTL studies and association mapping experiments is likely impossible for materials with different origins/gene pools. Most of the studies involving traditional QTL mapping have involved genetically distant cultivars/breeding lines as parents for the mapping populations to obtain the desired segregation for the studied trait in the progeny (Agarwal et al. 2008). Researchers have used parent genotypes from different continents or even different species (e.g., wild and cultivated barley crossings) (Forster et al. 2000). This fact alone makes combining the results of most existing traditional QTL studies with association mapping results (which are mainly generated for related genotypes) difficult or perhaps even pointless. The procedure of choosing of the parent genotypes for our previous study was different (Wójcik-Jagła et al. 2013), making such a combination possible.

The data for traditional QTL and association mapping experiments have never been combined for parameters related to drought tolerance in spring barley. This approach represents an alternative to the integration of the mapping results in previous barley studies (Alsop et al. 2011; Szűcs et al. 2009). Our experimental approach combines two independent genetic analyses of drought tolerance conducted with two sets of barley accessions. The use of DArTseq and SNP markers for mapping regions with QTLs considered responsible for drought tolerance has enabled a more detailed characterization of the drought response-related regions of the barley genome.

Although the most common approach to association mapping is genome-wide association study (GWAS), for the sake of our study and its practical aim of saturating already existing QTL regions related to drought tolerance in barley, we performed association mapping limited to those regions. A similar approach, resulting from a need to search for associations within previously selected candidate genes, was used by several researchers (Harjes et al. 2008; Zheng et al. 2008; Holliday et al. 2010; Caniato et al. 2014). Immense number of markers obtained only for the chosen regions and wide chromosomal coverage of these QTLs (Wójcik-Jagła et al. 2013) were enough to determine the population’s structure and marker–trait associations.

The obtained population’s structure was related to some of the drought-tolerance parameters. One of the probable reasons for this is that the selection of the plant material for the association study was done by means of markers proposed in Fiust et al. (2015). The subpopulations obtained by STRUCTURE were not consistent with the criteria used for selecting the mapping population, though. The first and third subpopulation contained majority of tolerant or susceptible genotypes (according to the marker system only), respectively, but the second subpopulation consisted of the same number of both. The second possible reason of this correlation is the narrowing down of the set of DArTseq and SNP markers to those from the vicinity of already existing QTLs related to drought. However, out of three subpopulations revealed in this study there is none that is clearly the most or least tolerant in all aspects (physiological parameters measured) making it impossible to state that the population’s structure results from drought tolerance or one/few drought tolerance-related traits directly. In most of the studies involving association mapping population’s structure was established using markers of whole genome coverage (e.g., Jin et al. 2010; Chhatre et al. 2013; Gupta et al. 2014), even when the AM itself was not genome-wide (Harjes et al. 2008; Zheng et al. 2008; Holliday et al. 2010; Caniato et al. 2014). Yet, none of these studies aimed to complement and confirm results from a previous QTL study. In the light of the purpose of this study population’s structure that is related to drought tolerance to a certain extent is understandable and even favorable. All in all the mapping population was selected to be as drought tolerance-related as possible to reveal more drought response-related chromosome regions.

Our results indicate that linkage group 2H contains the highest number of significant associations. The markers located near the *QWC.sthf*-*2H* region flanked by the bPb-8949 and bPb-1967 markers, as well as the *Qqp.sthf*-*2H* and *QPSII.sthf*-*2H* regions (Wójcik-Jagła et al. 2013) are mostly associated with chlorophyll *a* fluorescence parameters. Chromosome 2H was confirmed as important for drought responses by Guo et al. (2008) based on changes in chlorophyll *a* fluorescence parameters. They confirmed that chromosome 2H carries two QTL regions for chlorophyll *a* fluorescence parameters. Other studies reported the presence of additional QTL regions for these parameters on barley chromosomes 3H, 4H, 5H, and 6H (Guo et al. 2008; Wójcik-Jagła et al. 2013).

Chlorophyll fluorescence measurements are more sensitive than other data for determining plant stress responses, which is why we observed that most of the associations were for chlorophyll fluorescence parameters. This method can indirectly indicate the changes in photosynthetic performance induced by even subtle changes in water relations, nutrient availability, light, CO_2_, photoassimilate demand, and other relevant traits (Kalaji et al. 2014, 2016). The connections between changes in chlorophyll fluorescence and other more direct physiological parameters were easily detected in the present study. The results presented herein reveal the association of some markers with chlorophyll fluorescence parameters as well as with water relations-related parameters, especially on chromosomes 2H and 5H. These chromosomes were previously determined as related to RWC (Teulat et al. 2003; Fan et al. 2015), WC (Wójcik-Jagła et al. 2013), and wilting score (Fan et al. 2015). Additionally, the markers associated with chlorophyll fluorescence parameters in the present study are located nearby markers within previously detected QTL regions for water relations (Wójcik-Jagła et al. 2013). Markers 32711359 and 100005565 from linkage group 2H are associated with chlorophyll fluorescence and correspond to markers bPb-8949 and bPb-7160 related to WC. Additionally, a similar pattern exists on linkage group 5H for markers 3397170, 3929528, and 5245116, which are located near marker bPb-8589. In this study, we detected markers associated with water relations neighboring markers bPb-1967 and bPb-1813, namely 7749450 and 3255497 on linkage groups 2H and 5H, respectively. These markers are located within regions related to drought-induced changes to chlorophyll fluorescence.

A similar relationship exists for chlorophyll fluorescence and gas exchange parameters. Marker 3396302 on linkage group 2H is associated with transpiration efficiency, and is located near (± 0.14 cM) bPb-6048 and bPb-6280, which are related to chlorophyll fluorescence parameters. Marker 3271910 on linkage group 3H is associated with *P*_n_ and ΦCO_2_, and is mapped near (± 0.14 cM) bPb-4645, which is located within regions containing QTLs related to ABS/CS and ET.

The region on chromosome 3H with markers 3272729 and bPb-0709 may be particularly valuable. These two markers are related to WUE and *P*_n_ in our present and previous study (Wójcik-Jagła et al. 2013). These parameters represent the ability of plants to remain highly productive under drought conditions. Genotypes with high *P*_*n*_ and leaf WC under drought conditions are more tolerant to water deficit stress (Rapacz et al. 2010b). These genotypes exhibit enhanced osmotic adjustment (Robredo et al. 2007), which is a crucial factor influencing the diversity in drought tolerance among barley accessions (Blum 1989).

In the present study some alleles have proven to be associated with more than one trait. This suggests that those traits are probably correlated. Chen and Lübberstedt (2010) distinguish two reasons for such a situation (genic pleiotropy)—true pleiotropy or intragenic linkage of quantitative trait polymorphism (QTP) alleles. In both of those cases the effect can be either desirable or not with regard to the traits involved. If the marker associated with more than one trait is to be used in a breeding program it is crucial to investigate the effect of the allele on each of the traits (true pleiotropy) or the phase of QTP alleles (intragenic linkage) (Chen and Lübberstedt 2010).

One way of finding out if there is no negative linkage between the traits or repulsion of the alleles is the analysis of allele substitution effects (Würschum 2012). This kind of analysis can be used when the model of choice for the AM study is the mixed linear model (MLM) or its modifications that allow calculation of allele substitution effects (e.g., Liu et al. 2011; Würschum et al. 2011; Berger et al. 2013). However, even in the studies using models enabling such calculations, analysis of allele substitution effects is often not presented or discussed (e.g., Holliday et al. 2010; Sonah et al. 2015). In our study, the association mapping was performed using the GLM approach for the regions neighboring loci already known as involved in drought tolerance (Wójcik-Jagła et al. 2013). Neumann et al. (2011) in his study comparing GLM and MLM models for GWAS in bread wheat suggested that the GLM approach may be useful for comparing of the results with already known loci, especially as in some cases it is more sensitive than MLM (Yu et al. 2009).

Another approach to dissecting the nature of correlation of traits associated with one locus and the effect of the locus in question on each of them is performing big-scale field performance tests combined with identification of the presence/absence of the interesting marker loci. Such tests were performed for the markers used in this study for selection of the mapping population. We correlated the presence/absence of a marker with physiological traits and traits of agronomic importance (e.g., yield, protein content, height, etc.) (data not shown). This procedure, preceded by the necessary step of marker conversion (Fiust et al. 2015), can be used also for the markers associated with the most important physiological traits from this study.

Our results confirm some of the findings of an earlier QTL study on Polish spring barley materials (Wójcik-Jagła et al. 2013). The regions neighboring markers bPb-0572 and 3986215 (± 1.62 cM) on linkage group 2H (both related to RC/CS), 3267111 and bPb-6048, as well as bPb-6280 from *QPSII.sthf*-*2H* (± 0.53 cM; related to ΦPSII) verify that the results from published studies involving traditional QTL mapping can be compared with association mapping results even for different groups of plant materials. Additionally, these are examples of identical parameters that overlapped in two studies. However, other parameters that might differ describe the same processes and essentially provide the same information regarding the conditions of drought-stressed plants. For example, the association of marker 7749450 with WUE in the present study confirms the importance of the chromosome 2H region in the control of plant water relations in drought. Markers bPb-1967 (± 1.27 cM), bPb-7160 (± 4.82 cM), and bPb-8949 (± 6.38 cM) were mapped within a QTL region responsible for changes in leaf WC during drought stress. Furthermore, our data confirm that association mapping is useful for increasing the density of existing QTL maps without recreated mapping populations. Our study also reveals that the traditional biparental QTL mapping and association mapping results can be complementary when the analyses are conducted using materials from the same gene pool.

### Author contribution statement

MWJ and AF contributed to all the experimental process, data analysis and results interpretation, as well as paper preparing. JK coordinated phenotyping experiment and contributed to phenotyping data analysis. MR coordinated experimental process and contributed to the preparing of the final manuscript version.

## Electronic supplementary material

Below is the link to the electronic supplementary material.

Supplementary material 1 (PDF 52 kb)

Supplementary material 2 (XLSX 246 kb)
