# Supplementary material for: Association mapping of drought tolerance-related traits in barley to complement a traditional biparental QTL mapping study
Source: Theor Appl Genet. 2017 Oct 25;131(1):167–81. doi: 10.1007/s00122-017-2994-1 (PMC5750332; doi:10.1007/s00122-017-2994-1)
Supplement: Supplementary file 1 — Supplementary material 1 (PDF 52 kb) [file 122_2017_2994_MOESM1_ESM.pdf]

Journal: Theoretical and Applied Genetics

Association mapping of drought tolerance-related traits to complement a traditional biparental QTL mapping study

Magdalena Wójcik-Jagła*, Anna Fiust, Janusz Kościelniak, Marcin Rapacz

University of Agriculture in Krakow, Department of Plant Physiology, Podłużna 3, 30-239 Kraków, Poland

* Email: magdalena.p.wojcik@gmail.com, phone: +48 12 425 33 01

Online resource 1. Genotypes used in the study.

no	Genotype name	Type	Origin	Drought tolerance
1	DB06145-98	Advanced breeding line	HR Danko Ltd.	Tolerant
2	DM4832/11	Advanced breeding line	HR Danko Ltd.	Tolerant
3	DM4188/11	Advanced breeding line	HR Danko Ltd.	Tolerant
4	STH35004	Advanced breeding line	HR Strzelce Ltd. group IHAR	Tolerant
5	STH35021	Advanced breeding line	HR Strzelce Ltd. group IHAR	Tolerant
6	STH35026	Advanced breeding line	HR Strzelce Ltd. group IHAR	Tolerant
7	STH240	DH line	HR Strzelce Ltd. group IHAR	Tolerant
8	DM3494/09	Advanced breeding line	HR Danko Ltd.	Tolerant
9	DM2847/10	Advanced breeding line	HR Danko Ltd.	Tolerant
10	DM4479/11	Advanced breeding line	HR Danko Ltd.	Tolerant
11	STH35007	Advanced breeding line	HR Strzelce Ltd. group IHAR	Tolerant
12	STH35023	Advanced breeding line	HR Strzelce Ltd. group IHAR	Tolerant
13	STH34942	Advanced breeding line	HR Strzelce Ltd. group IHAR	Tolerant
14	STH393	DH line	HR Strzelce Ltd. group IHAR	Tolerant
15	DM3049/10	Advanced breeding line	HR Danko Ltd.	Tolerant
16	J08002/5	Advanced breeding line	HR Danko Ltd.	Tolerant
17	DM4480/11	Advanced breeding line	HR Danko Ltd.	Tolerant
18	STH35010	Advanced breeding line	HR Strzelce Ltd. group IHAR	Tolerant
19	STH35024	Advanced breeding line	HR Strzelce Ltd. group IHAR	Tolerant
20	STH470	DH line	HR Strzelce Ltd. group IHAR	Tolerant
21	STH391	DH line	HR Strzelce Ltd. group IHAR	Tolerant
22	J08002/10	Advanced breeding line	HR Danko Ltd.	Tolerant
23	DM4516/11	Advanced breeding line	HR Danko Ltd.	Tolerant
24	DM2682/10	Advanced breeding line	HR Danko Ltd.	Tolerant
25	STH35011	Advanced breeding line	HR Strzelce Ltd. group IHAR	Tolerant
26	STH35029	Advanced breeding line	HR Strzelce Ltd. group IHAR	Tolerant
27	STH366	DH lline	HR Strzelce Ltd. group IHAR	Tolerant
28	Suweren	Registered cultivar	HR Strzelce Ltd. group IHAR	Tolerant
29	DM2495/10	Advanced breeding line	HR Danko Ltd.	Tolerant
30	OLYMPIC	Registered cultivar	HR Danko Ltd.	Tolerant
31	DM3084/10	Advanced breeding line	HR Danko Ltd.	Tolerant
32	STH35014	Advanced breeding line	HR Strzelce Ltd. group IHAR	Tolerant
33	STH35031	Advanced breeding line	HR Strzelce Ltd. group IHAR	Tolerant
34	STH373	DH line	HR Strzelce Ltd. group IHAR	Tolerant
35	J09038/14	Advanced breeding line	HR Danko Ltd.	Susceptible
36	DB07022/1	Advanced breeding line	HR Danko Ltd.	Tolerant

37	DM2685/10	Advanced breeding line	HR Danko Ltd.	Tolerant
38	STH33562	Advanced breeding line	HR Strzelce Ltd. group IHAR	Tolerant
39	STH35017	Advanced breeding line	HR Strzelce Ltd. group IHAR	Tolerant
40	STH33570	Advanced breeding line	HR Strzelce Ltd. group IHAR	Tolerant
41	STH497	DH line	HR Strzelce Ltd. group IHAR	Tolerant
42	J09046/20	Advanced breeding line	HR Danko Ltd.	Susceptible
43	DM4474/11	Advanced breeding line	HR Danko Ltd.	Tolerant
44	DB07022/11	Advanced breeding line	HR Danko Ltd.	Tolerant
45	STH34999	Advanced breeding line	HR Strzelce Ltd. group IHAR	Tolerant
46	STH35019	Advanced breeding line	HR Strzelce Ltd. group IHAR	Tolerant
47	STH33575	Advanced breeding line	HR Strzelce Ltd. group IHAR	Tolerant
48	STH464	DH line	HR Strzelce Ltd. group IHAR	Tolerant
49	SOLDO	Registered cultivar	HR Danko Ltd.	Tolerant
50	DB07080/6	Advanced breeding line	HR Danko Ltd.	Tolerant
51	STH35003	Advanced breeding line	HR Strzelce Ltd. group IHAR	Tolerant
52	STH35020	Advanced breeding line	HR Strzelce Ltd. group IHAR	Tolerant
53	STH33598	Advanced breeding line	HR Strzelce Ltd. group IHAR	Tolerant
54	STH472	DH line	HR Strzelce Ltd. group IHAR	Tolerant
55	DM4690/11	Advanced breeding line	HR Danko Ltd.	Susceptible
56	J08060/2	Advanced breeding line	HR Danko Ltd.	Susceptible
57	J09003/14	Advanced breeding line	HR Danko Ltd.	Susceptible
58	STH33846	Advanced breeding line	HR Strzelce Ltd. group IHAR	Susceptible
59	STH34936	Advanced breeding line	HR Strzelce Ltd. group IHAR	Susceptible
60	STH34874	Advanced breeding line	HR Strzelce Ltd. group IHAR	Susceptible
61	STH156	DH line	HR Strzelce Ltd. group IHAR	Susceptible
62	DB07117/4	Advanced breeding line	HR Danko Ltd.	Susceptible
63	J08060/16	Advanced breeding line	HR Danko Ltd.	Susceptible
64	J09008/3	Advanced breeding line	HR Danko Ltd.	Susceptible
65	STH33320	Advanced breeding line	HR Strzelce Ltd. group IHAR	Susceptible
66	STH34984	Advanced breeding line	HR Strzelce Ltd. group IHAR	Susceptible
67	STH34875	Advanced breeding line	HR Strzelce Ltd. group IHAR	Susceptible
68	STH491	DH line	HR Strzelce Ltd. group IHAR	Susceptible
69	DM2632/10	Advanced breeding line	HR Danko Ltd.	Susceptible
70	J08062/3	Advanced breeding line	HR Danko Ltd.	Susceptible
71	J09011/14	Advanced breeding line	HR Danko Ltd.	Susceptible
72	STH33424	Advanced breeding line	HR Strzelce Ltd. group IHAR	Susceptible
73	STH34985	Advanced breeding line	HR Strzelce Ltd. group IHAR	Susceptible
74	STH34876	Advanced breeding line	HR Strzelce Ltd. group IHAR	Susceptible
75	STH293	DH line	HR Strzelce Ltd. group IHAR	Susceptible
76	DM2968/10	Advanced breeding line	HR Danko Ltd.	Susceptible
77	J08084/3	Advanced breeding line	HR Danko Ltd.	Susceptible
78	J09018/9	Advanced breeding line	HR Danko Ltd.	Susceptible
79	STH33425	Advanced breeding line	HR Strzelce Ltd. group IHAR	Susceptible
80	STH34818	Advanced breeding line	HR Strzelce Ltd. group IHAR	Susceptible
81	STH34878	Advanced breeding line	HR Strzelce Ltd. group IHAR	Susceptible
82	STH294	DH line	HR Strzelce Ltd. group IHAR	Susceptible
83	J08005/19	Advanced breeding line	HR Danko Ltd.	Susceptible
84	J08084/20	Advanced breeding line	HR Danko Ltd.	Susceptible
85	J09034/10	Advanced breeding line	HR Danko Ltd.	Susceptible
86	STH34838	Advanced breeding line	HR Strzelce Ltd. group IHAR	Susceptible
87	STH34819	Advanced breeding line	HR Strzelce Ltd. group IHAR	Susceptible
88	STH34880	Advanced breeding line	HR Strzelce Ltd. group IHAR	Susceptible
89	STH301	DH line	HR Strzelce Ltd. group IHAR	Susceptible
90	J08008/17	Advanced breeding line	HR Danko Ltd.	Susceptible
91	J08085/6	Advanced breeding line	HR Danko Ltd.	Susceptible
92	J09043/3	Advanced breeding line	HR Danko Ltd.	Susceptible
93	STH34851	Advanced breeding line	HR Strzelce Ltd. group IHAR	Susceptible
94	STH34821	Advanced breeding line	HR Strzelce Ltd. group IHAR	Susceptible
95	STH34883	Advanced breeding line	HR Strzelce Ltd. group IHAR	Susceptible
96	STH330	DH line	HR Strzelce Ltd. group IHAR	Susceptible
97	J08055/16	Advanced breeding line	HR Danko Ltd.	Susceptible

98	J08085/10	Advanced breeding line	HR Danko Ltd.	Susceptible
99	STH34991	Advanced breeding line	HR Strzelce Ltd. group IHAR	Susceptible
100	STH34903	Advanced breeding line	HR Strzelce Ltd. group IHAR	Susceptible
101	STH34822	Advanced breeding line	HR Strzelce Ltd. group IHAR	Susceptible
102	STH34959	Advanced breeding line	HR Strzelce Ltd. group IHAR	Susceptible
103	STH483	DH line	HR Strzelce Ltd. group IHAR	Susceptible
104	J08056/10	Advanced breeding line	HR Danko Ltd.	Susceptible
105	J08086/6	Advanced breeding line	HR Danko Ltd.	Susceptible
106	STH34441	Advanced breeding line	HR Strzelce Ltd. group IHAR	Susceptible
107	STH34935	Advanced breeding line	HR Strzelce Ltd. group IHAR	Susceptible
108	STH34839	Advanced breeding line	HR Strzelce Ltd. group IHAR	Susceptible
109	STH34964	Advanced breeding line	HR Strzelce Ltd. group IHAR	Susceptible
